# Commentary on ‘Untargeted CUT&Tag reads are enriched at accessible chromatin and restrict identification of potential G4-forming sequences in G4-targeted CUT&Tag experiments’

**DOI:** 10.1093/nar/gkaf1337

**Published:** 2025-12-12

**Authors:** Larry Melidis, Rafael de Cesaris Araujo Tavares, Xuan He, Somdutta Dhir, David Tannahill, Shankar Balasubramanian

**Affiliations:** Yusuf Hamied Department of Chemistry, University of Cambridge, Cambridge, CB2 0RE, United Kingdom; Cancer Research UK Cambridge Institute, University of Cambridge, Cambridge, CB2 1EW, United Kingdom; Cancer Research UK Cambridge Institute, University of Cambridge, Cambridge, CB2 1EW, United Kingdom; Yusuf Hamied Department of Chemistry, University of Cambridge, Cambridge, CB2 0RE, United Kingdom; Cancer Research UK Cambridge Institute, University of Cambridge, Cambridge, CB2 1EW, United Kingdom; Cancer Research UK Cambridge Institute, University of Cambridge, Cambridge, CB2 1EW, United Kingdom; Yusuf Hamied Department of Chemistry, University of Cambridge, Cambridge, CB2 0RE, United Kingdom; Cancer Research UK Cambridge Institute, University of Cambridge, Cambridge, CB2 1EW, United Kingdom; School of Clinical Medicine, University of Cambridge, Cambridge, CB2 OSP, United Kingdom

In a recent study (Nucleic Acids Research, Volume 53, Issue 14, 12 August 2025, gkaf678), Thompson and Byrd examine potential issues in the mapping of DNA G-quadruplexes (G4s) by CUT&Tag and related methods [1]. The authors conclude that detection of G4s in chromatin is significantly confounded by artefacts arising from background Tn5 enzymatic activity independent of the primary G4-targeting probe. We highlight several concerns regarding the authors’ meta-analysis of published data, including the problematic use of computational tools developed initially for other experimental modalities, such as ATAC-seq and ChIP-seq, for G4-CUT&Tag data without explicit disclosure of the analytical methods and code. Given that CUT&Tag is widely used and the technology is rapidly evolving, it is timely to promote best practices in the profiling of G4s and other features.

A critical point is to clarify the inherent differences between G4-targeted and untargeted CUT&Tag libraries. CUT&Tag methods rely on the selective recognition of a molecular feature on chromatin by a high-affinity probe, followed by the recruitment of Tn5 transposase to insert adapters in the vicinity of the target [2] to prepare a sequencing library. This is performed under high salt conditions to suppress the otherwise high-affinity transposase binding (utilized in ATAC-seq) and reduce non-targeted tagmentation. Depending on the target, this creates an overall sparse distribution of mapped read fragments across the genome, with local enrichment around the target and minimal random tagmentation, compared to other methods.

**Figure 1.**
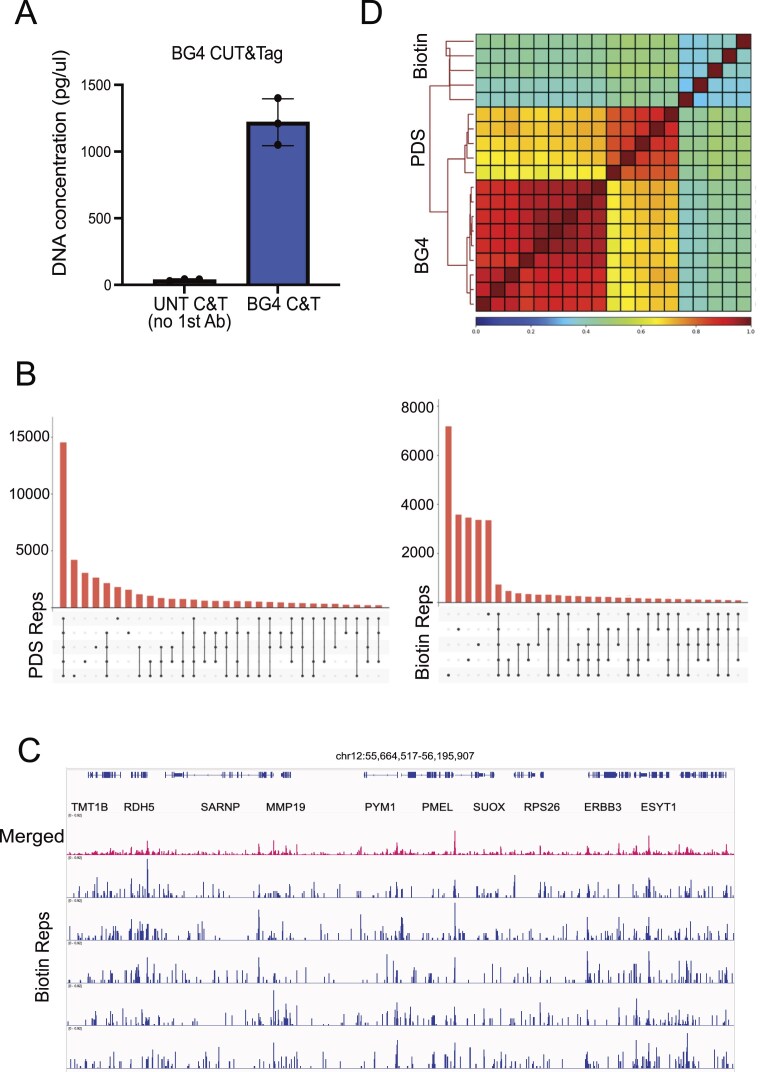
Material recovery, replicate reproducibility, and correlation analysis between G4-targeted and untargeted CUT&Tag experiments. (**A**) DNA concentration (Tapestation HS D1000) of untargeted and BG4 CUT&Tag libraries. Substantially more DNA is recovered when using a targeted (e.g. BG4) relative to untargeted (e.g. no primary antibody) libraries [9]. (**B**) Upset diagrams of SEACR *P* = .01 peaks for five replicates for Biotin Chem-map (untargeted control, right graph) and pyridostatin (PDS) Chem-map (G4-targeted experiment, left graph) [8]. The majority of peaks called from untargeted data (>90%) do not overlap across replicates, whereas >60% of peaks overlap in G4-targeted replicates. (**C**) Group-scaled genome browser tracks (bin size of 5, highest value 0.92, visualized with on IGV) for five independent replicates of untargeted (biotin) Chem-map data [8]. Individual replicates resulting from random tagmentation events are shown in blue. Merging replicates together (red track) reveals an apparent strong peak corresponding to a high Tn5-driven background site arising from noisy untargeted data (resulting in artificial background inflation). (**D**) Spearman correlation heatmap for biotin Chem-map, PDS Chem-map, and BG4 CUT&Tag libraries, across the genome with 500 bp bins. (Data from Chem-map [8] and independent BG4 published data [11] on K562 cells). High correlation (*P* = ~0.7–0.8) is observed between PDS and BG4 data, in contrast low correlation (*P* = ~0.4–0.5) is detected between BG4/PDS and biotin data. Importantly, replicate correlation across biotin datasets is poor (*P* = ~0.3) when compared to PDS/BG4 (*P* = ~0.9).

In the absence of a target, e.g. if the primary probe is omitted, or a non-specific antibody (e.g. serum IgG) is used in place of the primary probe, substantially less DNA is acquired in library preparation (Fig. 1A). Accurate quantification of those libraries is challenging, and when they are sequenced, this often leads to over-representation of polymerase chain reaction duplicates. This is a crucial point as it influences downstream analysis. The combination of targeted tagmentation with the sequence bias of the Tn5 transposase itself can create true duplicate fragments originating from independent molecular events. This has been explored in ATAC-seq experiments using UMIs [3], but not yet for CUT&Tag.

The primary aim of a CUT&Tag experiment is to identify regions where the feature resides through peak calling. Peak callers [4, 5] have been developed specifically for these experimental data sets, exploiting the typically sparse distribution of sequenced fragments in off-target regions. In a CUT&Tag experiment, identifying local enrichment obviates the need for an explicit background sample, since selective enrichment is experimentally absent. Bioinformatically, identification of enriched regions is achieved by introducing various statistical (AUC, *P*-value [3]) and absolute thresholds (number of fragments [4] within the same library, which are distinct from those used by ChIP-seq peak callers [6]; even in targeted experiments, this can be challenging [7]), as the peaks identified can vary slightly depending on sequencing depth and experimental variability. For these reasons, we consider consensus peaks across multiple replicates (both technical and biological) to reflect the most reliable output of G4 CUT&Tag or Chem-map experiments. This method also helps to discriminate between targeted and untargeted libraries. For example, in the recent development of Chem-map [8]), five technical replicates were performed for each biological replicate to avoid random peak calling. This demonstrated the noisy nature of peak calling in untargeted sequencing data (<10% of peaks overlap across multiple untargeted replicates, whereas >60% overlap in targeted replicates with the same number of replicates, Fig. 1B). For that reason, all subsequent analysis should be performed on the consensus peak set, including the fraction of reads in peaks (as defined by the consensus). As the assay output is based on Tn5 activity, any consensus peaks residually arising from untargeted libraries predominantly correspond to unsuppressed residual Tn5 background activity and will be found in open chromatin regions defined by Tn5-based ATAC-seq experiments. Given G4 structures are primarily features of open chromatin [9, 10], the reported peak co-localization between untargeted versus G4-targeted experiments is unsurprising and inevitable. The key issue is whether the signal obtained in the presence of a G4 probe can be confidently distinguished from the tagmentation background naturally occurring in its absence.

Quantification of sequencing results is not a straightforward process and is only possible under a series of prerequisites (same number of cells, parallel experiments with the same concentrations and reagents, etc.). Merging or averaging mapped reads from multiple experiments should be strongly discouraged as it can lead to artefacts like an apparent enrichment of background over noise even in untargeted libraries (Fig. 1C). This can lead to the false conclusion that background levels are similar to targeted signal. The differential read accumulation within peaks between two conditions can sometimes be addressed using DiffBind (https://bioconductor.org/packages/devel/bioc/vignettes/DiffBind/inst/doc/DiffBind.pdf), which was developed for differential binding analysis on ChIP-seq read counts in genomic regions (peaks). However, DiffBind utilizes DESeq2 or edgeR, both developed for RNA-seq analysis and assumes the total messenger RNA material is similar across the conditions. In ChIP-seq, comparable DNA recovery and sequencing depths are usually obtained. In contrast to CUT&Tag, peaks in ChIP-seq are defined by the relative enrichment of read counts in the target sample over the input sample for each genomic region; thus, normalizing by read count across the genome is sound statistical practice. For CUT&Tag, the conditions for similar DNA recovery and read distributions between samples do not generally hold when comparing targeted and untargeted libraries; therefore, it is our view that normalization methods within these packages are unsuitable, including DESeq2-mediated background bin normalization and RLE used in Thompson and Byrd [1]. However, DiffBind could be applied when comparing targeted libraries that use the same probe for different cell conditions, such as small perturbations that result in similar DNA recovery. The extreme difference in levels of DNA recovery between targeted and non-targeted probes challenges the limits of normalization within DiffBind and makes this type of comparison between untargeted and G4-targeted libraries wholly unsuitable.

A further consideration is the application of complexity normalization between the untargeted and targeted CUT&Tag libraries. As CUT&Tag is fundamentally different from ChIP-, ATAC-, or RNA-seq, down-sampling or merging of sparsely distributed reads skews the real signal distribution and creates artefacts (Fig. 1C). In fact, the difference in DNA recovery between targeted and untargeted libraries, along with fragment length distribution is a biologically meaningful measure that forms part of the experimental result, i.e. a high-affinity probe selectively recovers more DNA from specific genomic features relative to noise or non-specific controls. This can be seen in the correlation between replicates genome-wide with bins of 500 bp (Fig. 1D).

Finally, as for any chromatin profiling experiment, G4 CUT&Tag should be interpreted within the experimental context and the biological question under investigation. For genome-wide G4 profiling, we recommend that multiple biological and technical replicates (at least three for each but preferably more) are used to determine the set of robust consensus peaks. Furthermore, as peak calling is highly threshold- and algorithm-dependent, we recommend using only the highly reproducible peak set for downstream analyses, with appropriate and justified computational tools, alongside extension of reads and small bin size <10 for visualization. When investigating larger perturbations in genomic landscapes between conditions, we suggest using suitable fiducials, such as Drosophila S2 genomic DNA, cells, and/or synthetic G4 template spike-ins, to facilitate accurate quantification of G4 signal differences between samples.
